# Endoleak and Pseudoaneurysm Formation in the Setting of Stent Graft Infection Following Endovascular Uretero-Arterial Fistula Repair: The Dreaded Complication

**DOI:** 10.7759/cureus.8830

**Published:** 2020-06-25

**Authors:** Abby L Perrenoud, Garret Heiberger, Jackson Shriver, Douglas Yim

**Affiliations:** 1 Interventional Radiology, University of South Dakota Sanford School of Medicine, Sioux Falls, USA; 2 Interventional Radiology, Avera McKennan Hospital and University Health Center, Sioux Falls, USA; 3 Medicine/Radiology, University of South Dakota Sanford School of Medicine, Sioux Falls, USA

**Keywords:** ureteroarterial fistula, pseudoaneurysm, stent graft infection, endoleak

## Abstract

The complication of uretero-arterial fistula after prolong ureteral stenting is well recognized. The treatment is primarily endovascular stenting across the fistulous communication accepting the potential risk of stent graft infection. Herein we present a case of a 71-year-old female who developed an uretero-arterial fistula after prolong ureteral stenting and exchanges following ileal conduit obstruction. Initial treatment with left common iliac stenting controlled the hematuria, but only temporarily. Repeat angiography revealed a type 1b endoleak requiring stent extension. Unfortunately, persistent hematuria necessitating further angiography showed the development of a saccular pseudoaneurysm around the stent graft requiring proximal stent extension. A nuclear medicine indium 111-tagged white blood cell scan with single-photon emission CT (SPECT)/CT confirmed stent graft infection. Conservative therapy with antibiotics failed, causing graft failure that ultimately required bypass surgery.

## Introduction

Uretero-arterial fistula (UAF) is a rare but potentially life-threatening complication that should be considered in patients presenting with intermittent gross hematuria and a history of pelvic surgery/radiation, ureteral manipulation and chronic cannulation [1,2]. UAF arises where the cannulated ureter and the common or external iliac artery overlap. Its development can be traced through the pulsation of the artery and the pressure on the ureter [3]. The median delay between surgery and hematuria is roughly two years for the patients with malignant disease. Although mortality rates in the 1980s were approximately 70%, improvements in diagnosis and management have reduced it to approximately 23% [4-7].

## Case presentation

**Figure 1:**
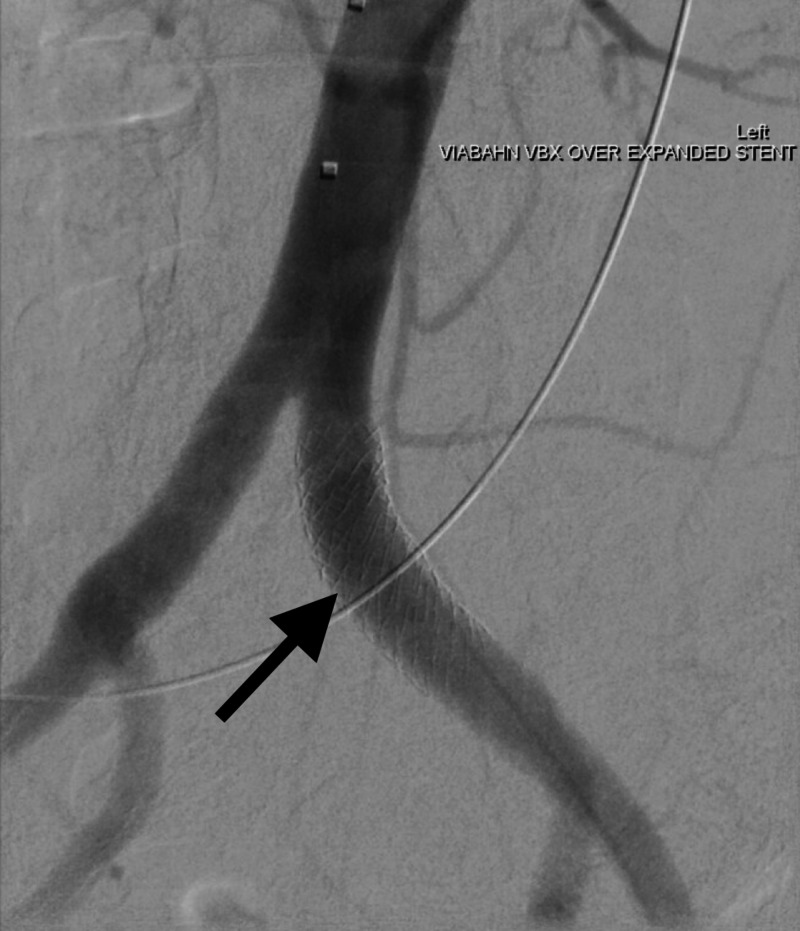
Endovascular stent within the lumen of the left iliac artery. No flow through the ureteroarterial fistula was noted.

A 71-year-old female with a significant history of endometrial and vaginal cancer ultimately underwent exenteration with ileal conduit reconstruction after radiation and chemotherapy. She developed a left ureteral-ileal anastomotic stricture, ultimately requiring a retrograde transileal nephroureteral stent (RNUS). During a routine exchange of the RNUS, profound arterial bleeding was noted in the ileal conduit. Immediate angiography confirmed a suspected UAF where the left ureter overlapped the left common iliac artery. Hemostasis was obtained following left common iliac artery stent graft placement across the fistula. RNUS stent exchange was then completed successfully (Figure 1).

Within a week, hematuria represented, and the patient became febrile. Blood and urine cultures grew Klebsiella oxytoca, for which she received intravenous antibiotics. Repeat left iliac arteriogram revealed a type 1b endoleak from the distal stent edge, which resolved with iliac stent extension. The RNUS stent was exchanged, and she was later discharged in a stable condition on a long course of antibiotics.

**Figure 2:**
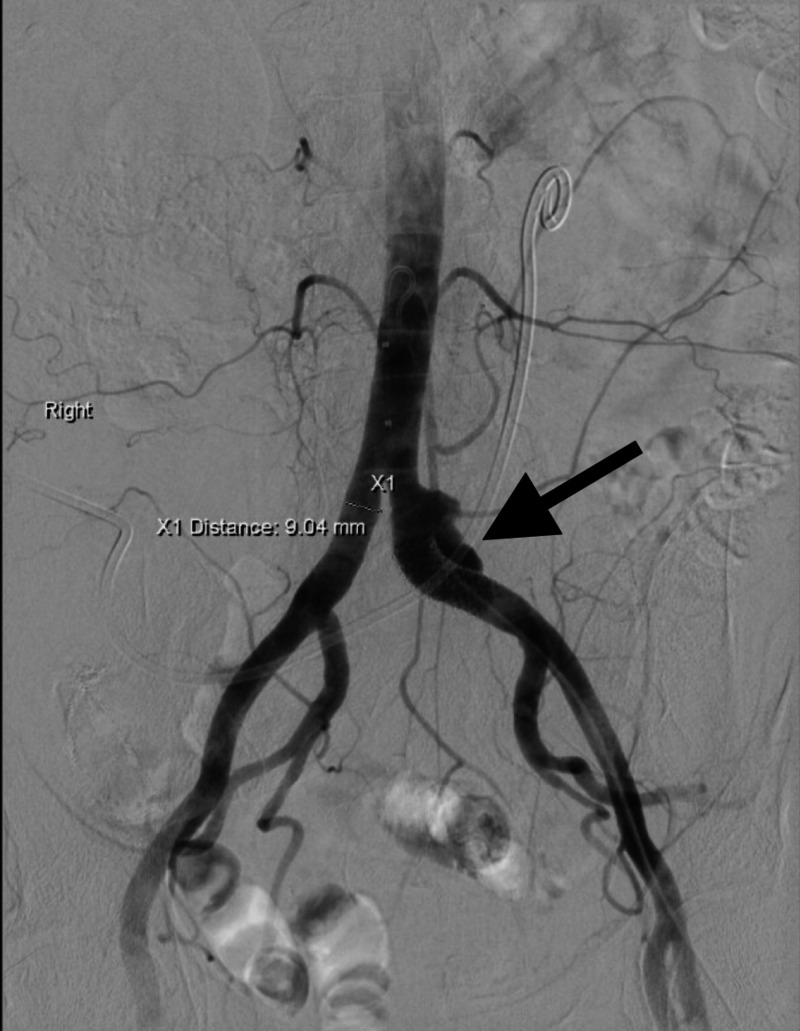
Iliac arteriogram demonstrating wide mouth pseudoaneurysm 1 cm proximal to the left common iliac artery origin proximal to the stent graft with active hemorrhage.

**Figure 3:**
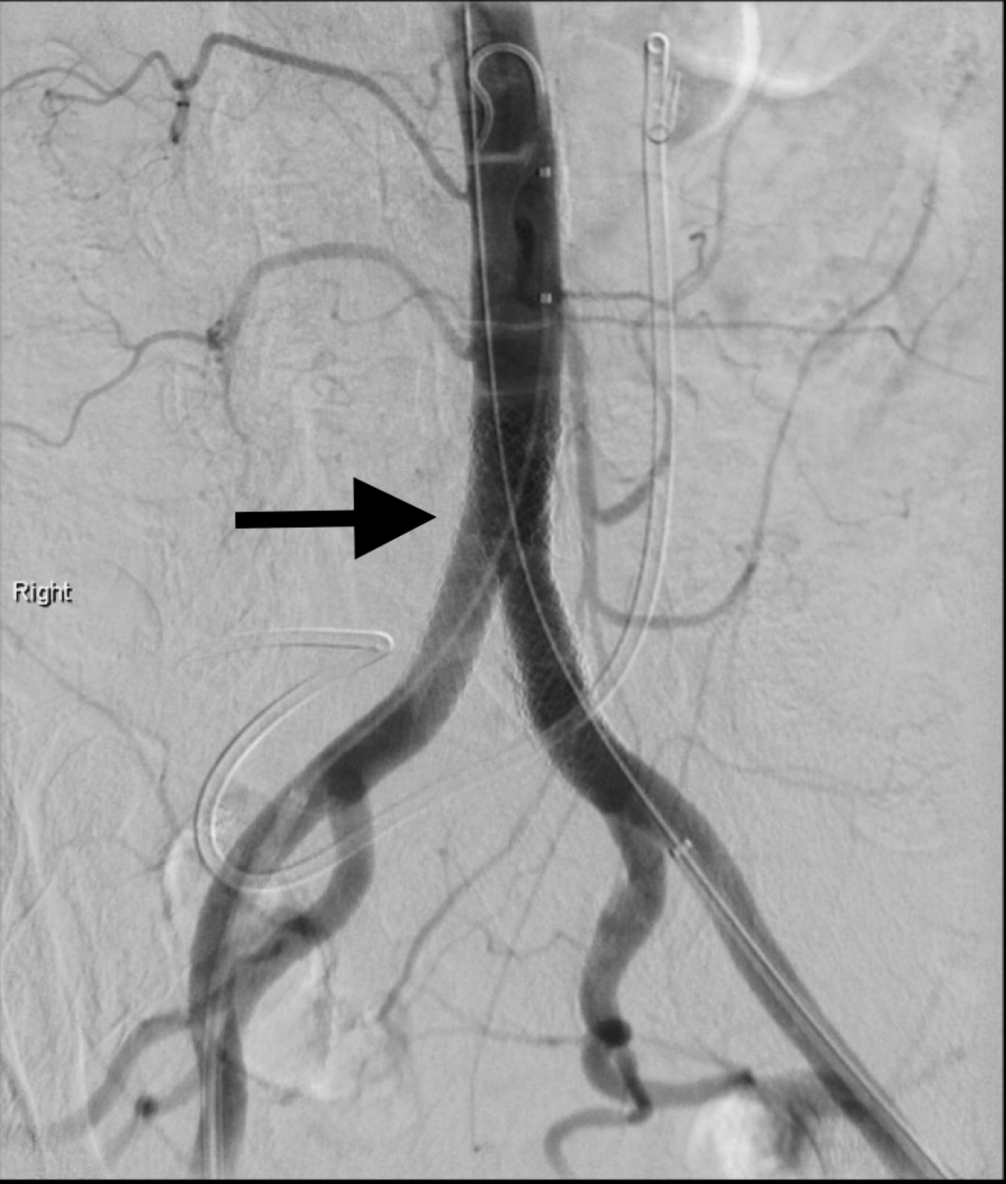
Iliac arteriogram illustrating bilateral kissing common iliac artery stent grafts.

Four days later, she presented with decreased urine output and recurrent bleeding from her stoma. During admission, the RNUS stent became dislodged causing copious exsanguination from her ostomy site. Angiography revealed a saccular pseudoaneurysm 1 cm proximal to the left common iliac artery stent graft (Figure 2), necessitating further extension with the placement of bilateral kissing iliac stent grafts (Figure 3) to exclude the pseudoaneurysm. Additionally, the RNUS stent was removed and bilateral nephrostomy tubes were placed to divert the urine.

**Figure 4:**
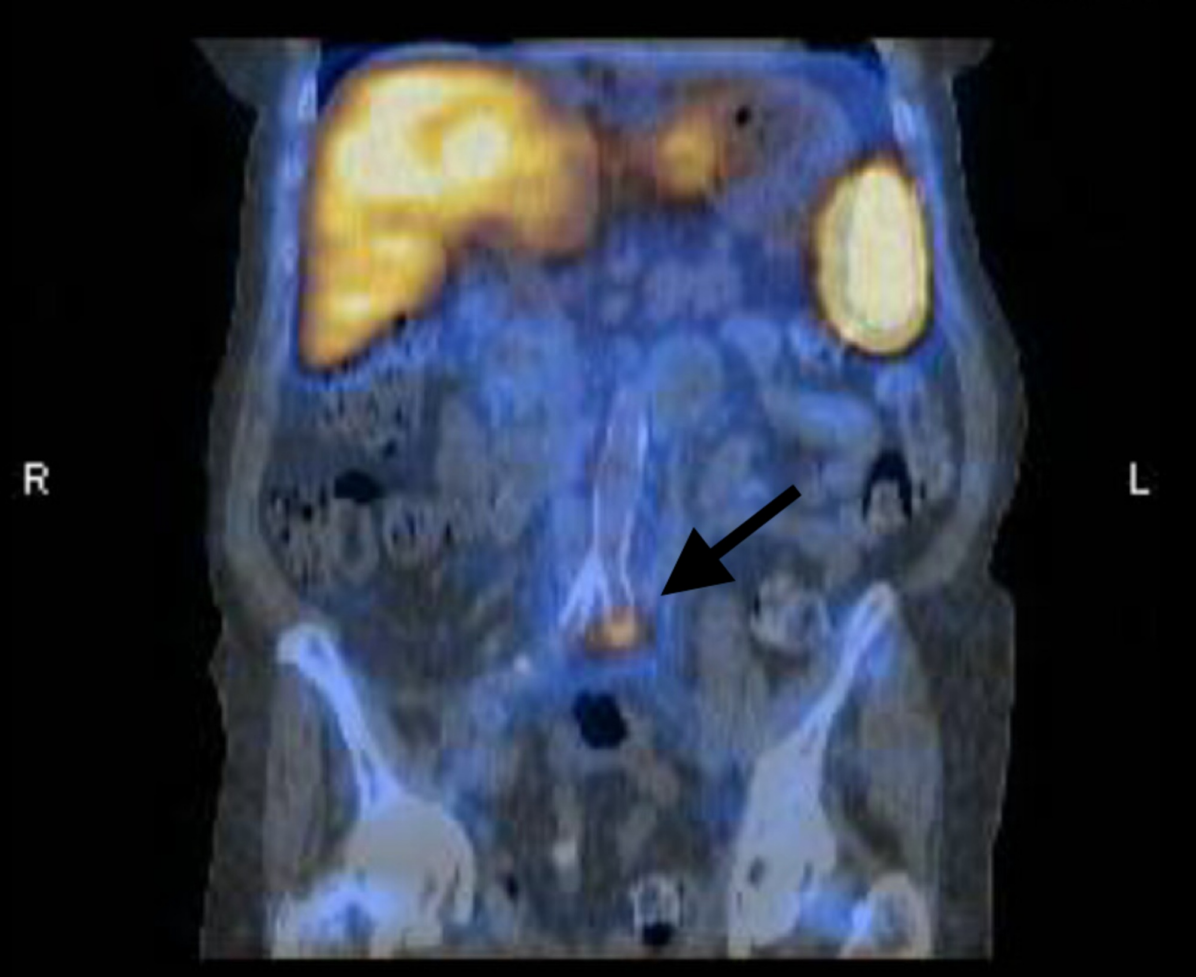
Focal radiolabeled white blood cell localization of the left common iliac artery stent suspicious for infection.

A nuclear medicine indium 111 white blood cell scan with single-photon emission CT (SPECT)/CT was performed, which showed focal, radiolabeled white blood cell localization involving the left common iliac artery stent, suspicious for indolent infection (Figure 4). Despite suppressive oral antibiotics, she continued to have intermittent hematuria and ultimately required surgical revision and bypass.

## Discussion

Currently, there is no gold standard for the management of UAF, although endovascular repair has become the preferred treatment. A majority of published cases report no long-term procedural-associated complications, although data are scarce [8-11]. Okada et al. reported long-term outcomes at a mean follow-up of 548 (range 35-1,386) days, showing mild hematuria in 36% (n=4) of his participants and one death from urosepsis 37 days post-operatively [12]. However, stent graft infection is a dreaded complication of endovascular procedures. Staphylococcus aureus, Streptococcus, and fungi are the most commonly cultured organisms [13-15]. Despite this, to our knowledge there are no reported cases of an infected iliac stent graft in the setting of UAF treatment and only a few instances of infected iliac stent grafts in general [16].

Complications of stent graft infections include aneurysms, sepsis, and graft failure. Conservative treatment of stent graft infections includes antibiotics, while surgical therapy usually involves stent removal with reconstruction and bypass [13,15,17]. Risk factors for infection after stent graft placement include the breach in sterility, multiple reinterventions, and complicating endoleaks [18]. Iliac artery stenting is an invasive procedure and breach of sterility may be more frequent in an angiographic suite than the operating room due to increased foot traffic. Long guidewires and catheters are also prone to contamination when not securely handled. Furthermore, multiple and recurrent stent placement may increase the likelihood of infection [16].

Endoleaks are well documented and considered a risk factor for stent graft infection, so is having multiple reinterventions [18,19]. Primary endoleaks occur within 30 days of stent placement, and thereafter they are classified as secondary. Type I endoleak is caused by failure to achieve a circumferential seal at either the proximal (type 1a) or the distal end (type 1b) of the stent graft. Causes of primary type I endoleak include inappropriate anatomy, with a significantly angulated neck, significant calcification/plaque at the proximal or distal landing zone, a noncircular landing zone, malpositioning of the stent graft, type of endograft, and underdilation of the stent-graft. Secondary type I endoleak can be due to aneurysm remodeling, resulting in migration or progressive dilation of the proximal neck. In the setting of a type 1b endoleak due to an enlarged iliac artery or short stent landing, an extension endograft is necessary [20]. 

## Conclusions

This case highlights a rare but dreaded sequela (stent graft infection) of an equally rare complication (UAF). Strategies to avoid stent graft infection include maintaining sterile technique, appropriate antibiotic coverage, and mitigating procedural complications associated with infection (e.g. endoleaks). As endovascular repair is quickly becoming the preferred method for treatment of UAFs, awareness of this morbid outcome and strategies to best manage the risks becomes more prudent.
